# Effect of Maternal Age at Childbirth on Obesity in Postmenopausal Women: A Nationwide Population-Based Study in Korea

**DOI:** 10.1097/MD.0000000000003584

**Published:** 2016-05-13

**Authors:** Ji-Sun We, Kyungdo Han, Hyuk-Sang Kwon, Kicheol Kil

**Affiliations:** From the Department of Obstetrics and Gynecology (J-SW, KK), Yeouido St. Mary's Hospital; Department of Medical Statistics (KH), College of Medicine; and Division of Endocrinology and Metabolism (H-SK), Department of Internal Medicine, Yeouido St. Mary's Hospital, The Catholic University of Korea, Seoul, Korea.

## Abstract

The object of this study was to assess the obesity in postmenopausal women, according to age at childbirth.

We analyzed the association between age at first childbirth, age at last childbirth, parity, and subject obesity status (general obesity; BMI >25 kg/m^2^, nongeneral obesity; BMI ≤25 kg/m^2^, abdominal obesity; waist circumference >85 cm, nonabdominal obesity; waist circumference ≤85 cm), using data from a nationwide population-based survey, the 2010 to 2012 Korean National Health and Nutrition Examination Survey. Data from a total of 4382 postmenopausal women were analyzed using multivariate regression analysis with complex survey design sampling. And, the subjects were subdivided into groups according to obesity or not. Age, smoking, alcohol consumption, exercise, education, income level, number of pregnancies, oral contraceptive uses, breast feeding experience were adjusted as the confounders.

The prevalence of general obesity among Korean postmenopausal women was 37.08%. Women with general obesity and abdominal obesity were significantly younger at first childbirth compared with women with nongeneral obesity and no abdominal obesity (23.89 ± 0.1 vs. 23.22 ± 0.1, *P* <0.001). Age at first childbirth was inversely associated with obesity, while age at last childbirth was not associated with obesity or abdominal obesity. Women with a higher number of pregnancies were also more likely to have obesity and abdominal obesity. Age at first childbirth remained significantly associated with obesity, after adjusting for confounding factors.

Obesity in postmenopausal women is associated with first childbirth at a young age, and higher parity. Further research is needed to clarify the association between obesity and reproductive characteristics.

## INTRODUCTION

The prevalence of obesity varies between countries. Dramatic increases in the prevalence of obesity and its accompanying comorbidities are major health concerns in Korea. Lifestyle factors such as diet, insufficient physical activity, and adaptation to a Western lifestyle may be important causes of the rise in obesity. Currently, 32.8% of adults and 29.7% of women are estimated to be obese.^1,2^

Obesity increases the risk of cardiovascular disease, type 2 diabetes mellitus, dyslipidemia, metabolic syndrome, and obesity-related malignancies such as breast, colorectal, and other cancers.^3,4^ Obesity also elevates the risk of all-cause mortality. Abdominal obesity, in particular, is related more closely to undesirable outcomes than general obesity.

Obesity elderly is more common in women than in men. Weight gain during menopausal transition has been examined as a major contributing factor to body weight. Similarly, weight gain during pregnancy, and postpartum weight retention, have been identified as strong contributors to obesity.^5^ Pregnancy may promote weight gain, abdominal obesity, and metabolic diseases such as insulin resistance in later life. There is also a meaningful association between obesity and reproductive factors including early menarche, higher parity, and early motherhood.^6–9^

However, few reports have investigated the influence of age at first and last childbirths on obesity during menopause. The purpose of this study was to evaluate the relationship between the age of childbirth and obesity in postmenopausal women, using data from the 2010 to 2012 Korean National Health and Nutrition Examination Survey (KNHANES).

## METHODS

### Study Population and Data collection

This study is a cross-sectional population-based study. We analyze data from the 2010 to 2012 KNHANES, which is a nationwide, population-based, health examination survey performed regularly by the Division of Chronic Disease Surveillance of the Korea Centers for Disease Control and Prevention (the Korean Ministry of Health and Welfare). The KNHANES consists of a health interview, a health examination, and a nutrition survey. And, it is conducted by trained investigators. The survey was designed to monitor the health and nutritional status of the South Korean population.^10^ All of the participants signed an informed consent form prior to entering survey. For the survey performed between 2010 and 2012, target participants were selected from the 2009 National Census Registry using a stratified, multistage, probability sampling design that was based on geographic area, sex, and age. KNHANES uses a rolling sampling design; hence, the samples from each year are independent and homogeneous. Selected samples were weighted to ensure that they represented all noninstitutionalized female civilian populations in Korea. The Institutional Review Board of the Catholic University of Korea, Seoul, Korea, approved the use of the publicly available data for our statistical analyses.

Among those who participated in the survey in 2010 (n = 25,534), this study included 4546 postmenopausal women. We excluded data of women with no childbearing history and those missing data of reproductive and medical histories. Finally, 4382 women were included in this study. We divided participants into 4 groups for analysis: nongeneral obesity without abdominal obesity; nongeneral obesity with abdominal obesity; general obesity without abdominal obesity; and general obesity with abdominal obesity.

## SURVEY

### Measurement and Classification of Variables

The physical examination included height, body weight, and waist circumference (WC) using standard procedures. WC was measured midway between the costal margin and the iliac crest at the end of a normal expiration. Body mass index (BMI) was calculated using the formula BMI = weight (kg) ÷ height (m^2^).

Health interview for life style such as smoking status, alcohol consumption, exercise, education, income, and reproductive history such as parity, menopausal status, oral contraceptive use, hormone replacement therapy, age at first and last childbirths, were recorded by self-reported rating to questionnaires. Menopause was defined as having last menstrual period before more than 12 months. Regular exercise meant that the participant engages in moderate exercise for longer than 150 minutes per week, or severe exercise for longer than 60 minutes per week. Alcohol consumption was defined as drinking more than 5 g per day. Income level was stratified based on age and sex criteria, and the lowest income level corresponds to the lowest quartile of family income.

Obesity was defined as a BMI ≥25 kg/m^2^ in Asian participants.^1,11^ Abdominal obesity was defined as an waist circumference ≥85 cm, based on data from the Korean Society for the Study of Obesity.^1^

### Statistical Analysis

The baseline clinical characteristics of study group are analyzed by *T* tests and *χ*^2^ tests. Data are presented as means ± standard errors for continuous variables, and percentages ± standard errors for categorical variables. We analyzed associations between age at childbirth and obesity after adjustment for age, lifestyle factor, and reproductive factor using multivariate regression analysis. Statistical analyses were performed using SAS version 9.3 (SAS Institute, Cary, NC). A *P* value <0.05 was considered to be statistically significant.

## RESULTS

Our analysis included 4382 participants, all of whom were menopausal women. The mean age was 63.7 ± 0.3. The prevalence of general obesity (BMI >25 kg/m^2^) was 37.1%. The mean age at first and last childbirths was 23.7 ± 0.1 and 30.7 ± 0.1, respectively. Baseline characteristics of the study population are shown in Table 1, according to higher or lower BMI. There were differences between the study groups in educational level, parity, use of hormone replacement therapy, systolic and diastolic blood pressure, serum glucose level, serum and high-density cholesterol levels, and age at last childbirth (all *P* <0.05).

**TABLE 1:**
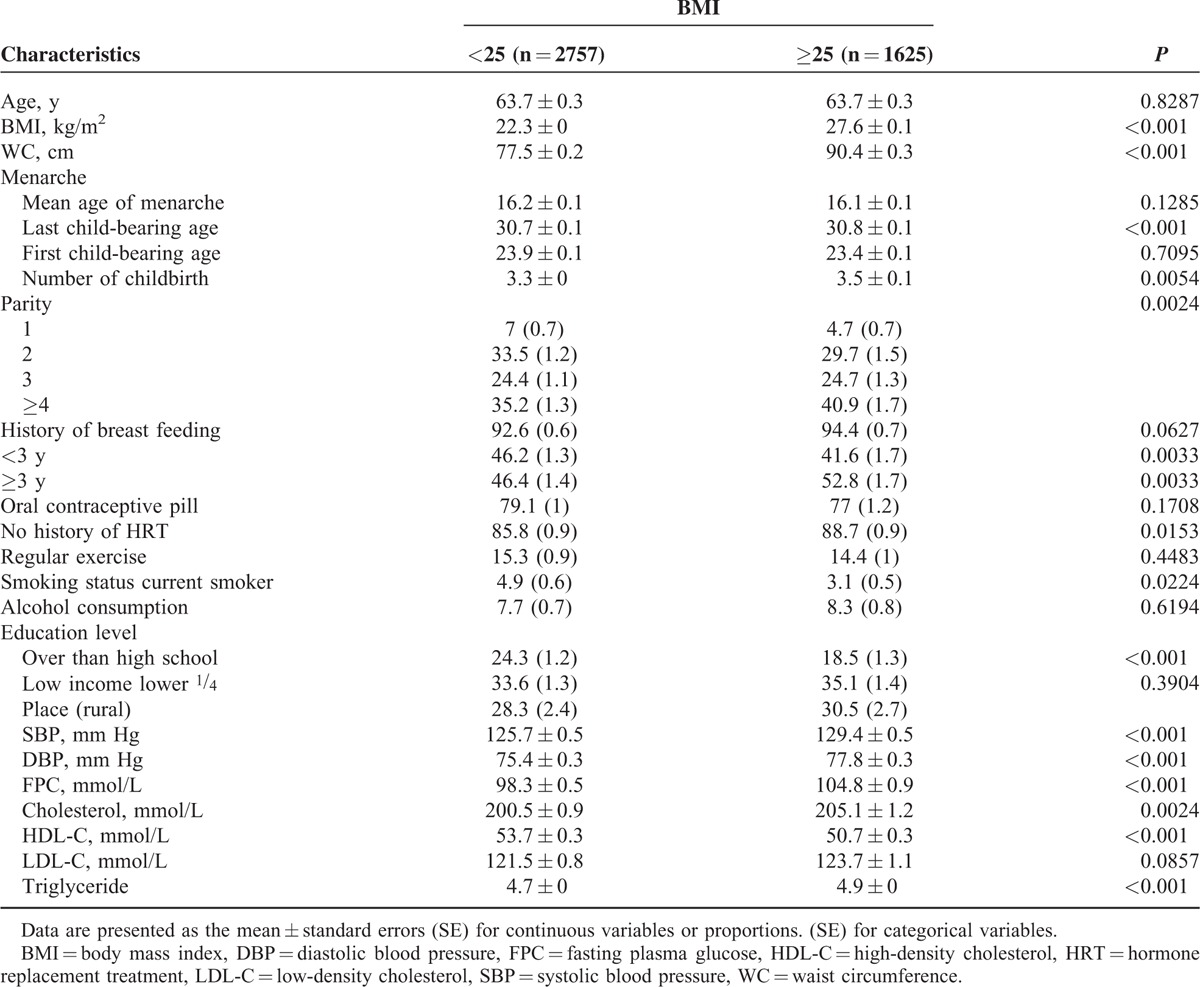
Baseline Clinical Characteristics of the Study Population

**FIGURE 1:**
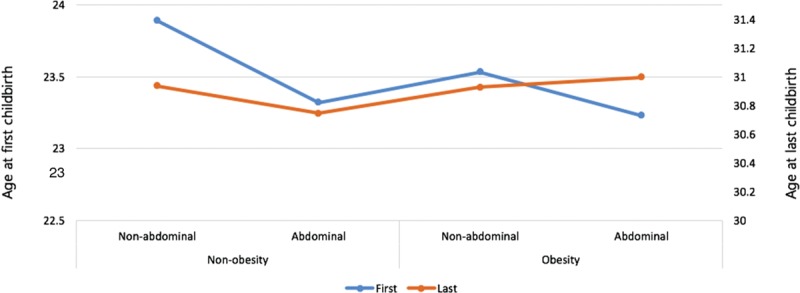
The mean age at first and last childbirths according to obesity categories. The group with general obesity and abdominal obesity had a significantly lower age at first childbirth compared with those with nongeneral obesity without abdominal obesity. Nonobesity BMI: <25 kg/m^2^; obesity BMI: ≥25 kg/m^2^. Nonabdominal obesity AC: <85 cm; abdominal obesity AC: ≥85 cm. BMI = body mass index, WC = waist circumference.

**FIGURE 2:**
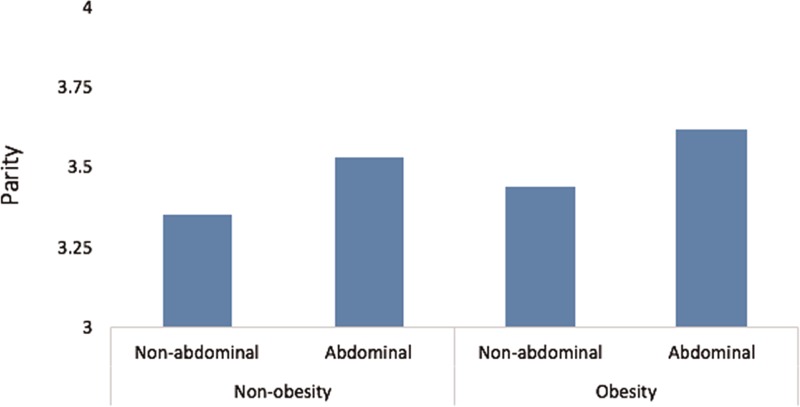
The mean parity according to obesity. A direct association is shown between parity and obesity, which is especially strong for abdominal obesity (*P* = 0.001). The prevalence of obesity was higher in women who had higher parity. Nonobesity BMI: <25 kg/m^2^; obesity BMI: ≥25 kg/m^2^. Nonabdominal obesity AC: <85 cm; abdominal obesity AC: ≥85 cm. BMI = body mass index, WC = waist circumference.

Figure 1 shows the comparisons of the mean age at first and last childbirths among the 4 groups. This analysis found that the group with general obesity and abdominal obesity had a significantly lower age at first childbirth compared with those with nongeneral obesity without abdominal obesity (23.2 ± 0.1 vs. 23.9 ± 0.1, *P* <0.001). Figure 2 shows a direct association between parity and obesity, which is especially strong for abdominal obesity (*P* = 0.001). The prevalence of obesity was higher in women who had higher parity.

We performed multivariate regression analysis to identify the association between obesity and age at childbirth (Table 2). Age at first childbirth was inversely associated with obesity. This finding did not change after adjustment for confounding factors. However, age at last childbirth was not associated with obesity.

**TABLE 2:**
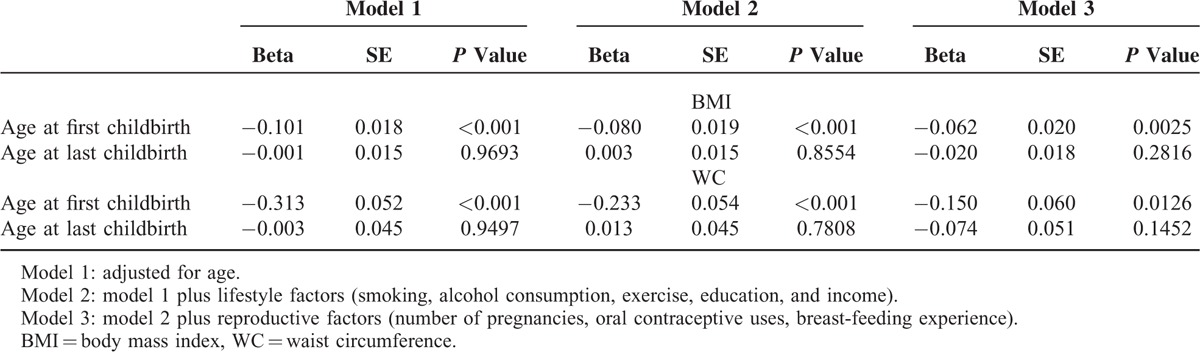
Multivariate Regression Analysis of Childbearing Age and Obesity

**FIGURE 3:**
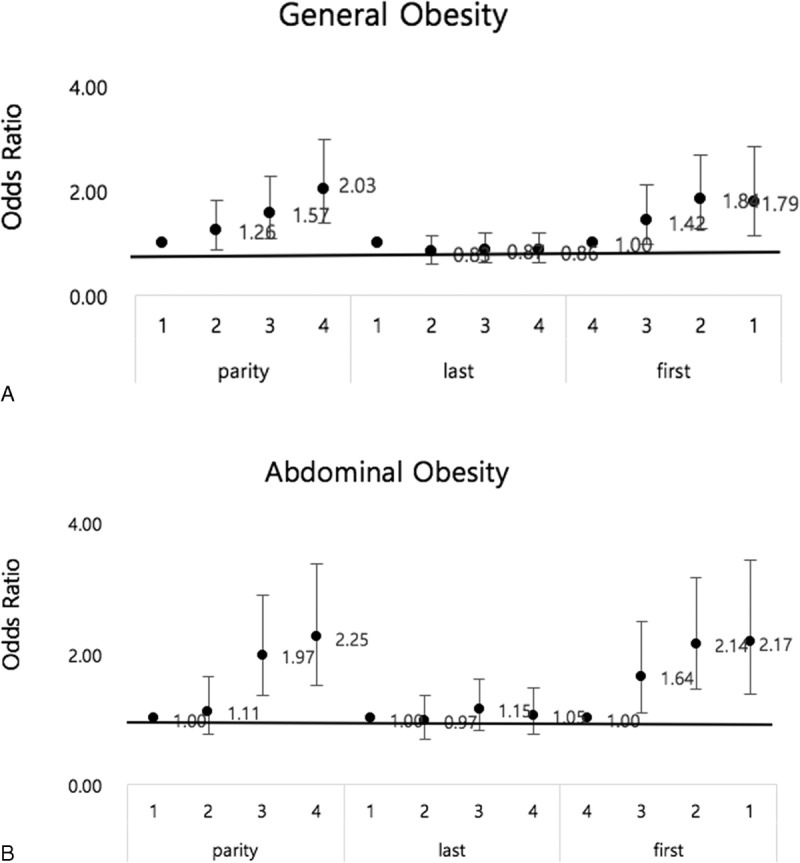
The relationship between obesity and maternal age or parity. (A) The graph shows a gradual increase in the risk of general obesity with the higher quartile of parity and with the lower quartile of age at first childbirth, but not in the age at last childbirth. (B) The graph shows a gradual increase in the risk of abdominal obesity with the higher quartile of parity and with lower quartile of age at first childbirth, but not in the age at last childbirth. Last, age at last childbirth; first, age at first childbirth; 1, 1st qurtile; 2, 2nd quartile; 3, 3rd quartile; 4, 4th quartile.

Higher parity and earlier age at first childbirth were significantly associated with both general and abdominal obesity (Figure 3). BMI increased progressively with increasing parity and decreasing age at first childbirth.

## DISCUSSION

This study demonstrates a relationship between age of childbirth and obesity in postmenopausal women, using data from the 2010 to 2012 KNHANES. The prevalence of general obesity and abdominal obesity was higher in women who were younger at first childbirth. This result remained after adjustment for confounding factors, including lifestyle factors and reproductive characteristics. Our results are consistent with a previous study that reported an association between age at first live birth and long-term weight gain in Chinese women 40 to 70-year old, although there were limitations due to the inclusion of both peri- and postmenopausal women in that study.^12^

The mechanisms for the positive association between obesity and earlier age at first childbirth are unknown. As this study had a cross-sectional design, it was not possible to determine which mechanisms connect younger age at first childbirth and increasing risk of obesity. There are several possible explanations for this mechanism.

First, weight retention caused by pregnancy and lifestyle change due to childrearing at early age may be the reason for obesity later life. Childbearing is associated with the development of overweight in women of reproductive age.^13^ Several studies indicated that approximately 13% to 20% of pregnant women experience substantial weight retention by 1 year postpartum, defined as body weight at least 5 kg above preconception weight.^14,15^ Possible mechanisms for this pattern included retention of gestational weight gain, sustained postpartum weight gain, dietary and lifestyle changes due to childbearing and child-rearing, behavioral and genetic factors influencing fat metabolism regulation, and hormonal changes.^16^ However, in KNHANES data we could not have information on obesity or not at prepregnancy, weight gain during pregnancy, postpartum weight retention, and lifetime weight change.

Second, earlier age at first childbirth may be causative of restriction of physical activity limited to nursing and housework is the possible explanation for obesity.^16^

Third, younger age at first childbirth is associated with increasing numbers of births.^17^ An increasing number of childbirth may have accumulated weight retention. These cumulative effects may contribute to obesity in later life. Most studies show that high parity is associated with weight gain and a higher risk of obesity or overweight.^18–20^ The mechanisms underlying the association between obesity and high parity are not known, but some evidence suggests that high maternal glucose, free fatty acid, and amino acid concentration may play a role in weight gain during pregnancy and postpartum weight retention, thereby increasing the risk of obesity in later life.^21,22^ Our results support an association between parity and obesity as well. Our study shows that adjustment for confounding factor including parity did not change the association between age at first childbirth and obesity in postmenopausal women.

Some studies have reported a relationship between number of childbirths and BMI, and a trend of greater upper body fat distribution with an increasing number of births.^16,17^ However, several studies have not found a direct relationship between parity and obesity.^21–25^ Martinez et al^21^ reported no relationship between the number of pregnancies and obesity. Koch et al^22^ found that parity was associated with BMI, but was not related to WC. Findings from another study indicated that women who had additional children did not gain more weight than primi-parous women did.^23^ Thus, there is no consensus about the association between parity and obesity.^5^

Our study shows an inverse relationship between age at first childbirth and obesity, and the association is stronger with abdominal obesity than general obesity. Earlier first childbirth was associated with increased abdominal obesity in both general and nongeneral obesity groups. The relationship with abdominal obesity was independent of age, recruitment site, education, and age at first full-term pregnancy.^21^ The mechanisms for the association between obesity and age at childbirth are not known; however, earlier age may result in restriction of physical activity to nursing and housework, thus providing a possible explanation for obesity. These findings are important because of the strong association with abdominal obesity, which has been identified as a risk factor for metabolic and cardiovascular diseases. We therefore conclude that first childbirth at an earlier age may be associated with poor health in menopausal women.

## LIMITATIONS

In many studies, obesity is defined as a BMI >30 kg/m^2^. However, most of Asian (Korean, Japan) is not obese. In Asian obesity was defined as a BMI >25 kg/m^2^, and the range for normal weight is between 18.5 and <25 kg/m^2^. There is a gap between this study and others in definition of term “obesity.” This study has other several limitations, including a lack of data about obesity during pregnancy, and the diet of included participants. However, this study has shown that earlier age at first childbirth and higher parity are risk factors for obesity in later life.

## CONCLUSION

We have shown that earlier age at first childbirth and higher parity are risk factors for obesity in later life. This study suggests that earlier age at first childbirth may be a risk factor for developing metabolic and cardiovascular diseases due to abdominal obesity. It is therefore important to educate mothers whose first childbirth occurs at an early age about the value of engaging in increased physical activity. And providing them with diet and nutritional education is also important. This data can be used broadly to design public health programs and support targeted health interventions for obese women before or after menopause. Health policymakers should develop appropriate strategies to decrease the incidence of obesity.
